# P-375. Real-World Comparison of BIC/TAF/FTC and DTG/TDF/FTC Regimens in ART-Naive HIV Patients: Virological, Immunological, and Metabolic Outcomes

**DOI:** 10.1093/ofid/ofaf695.593

**Published:** 2026-01-11

**Authors:** Yavuz Akcesme, Nurettin Erben, Saygın Nayman Alpat, Hasip kahraman, Elif Doyuk Kartal

**Affiliations:** Eskisehir Osmangazi University, Eskisehir, Eskisehir, Turkey; Eskisehir Osmangazi University, Eskisehir, Eskisehir, Turkey; Eskisehir Osmangazi University, Eskisehir, Eskisehir, Turkey; Eskisehir Osmangazi University, Eskisehir, Eskisehir, Turkey; Eskisehir Osmangazi University, Eskisehir, Eskisehir, Turkey

## Abstract

**Background:**

Although the widespread use of antiretroviral therapy has led to significant advances in HIV treatment, regimen selection still depends on factors such as efficacy, safety, and patient profile. This study aims to compare the virological, immunological, renal and metabolic outcomes of Bictegravir/Tenofovir Alafenamide/Emtricitabine and Dolutegravir/Tenofovir Disoproxil Fumarate/Emtricitabine regimens using real-world data.

**Figure:**
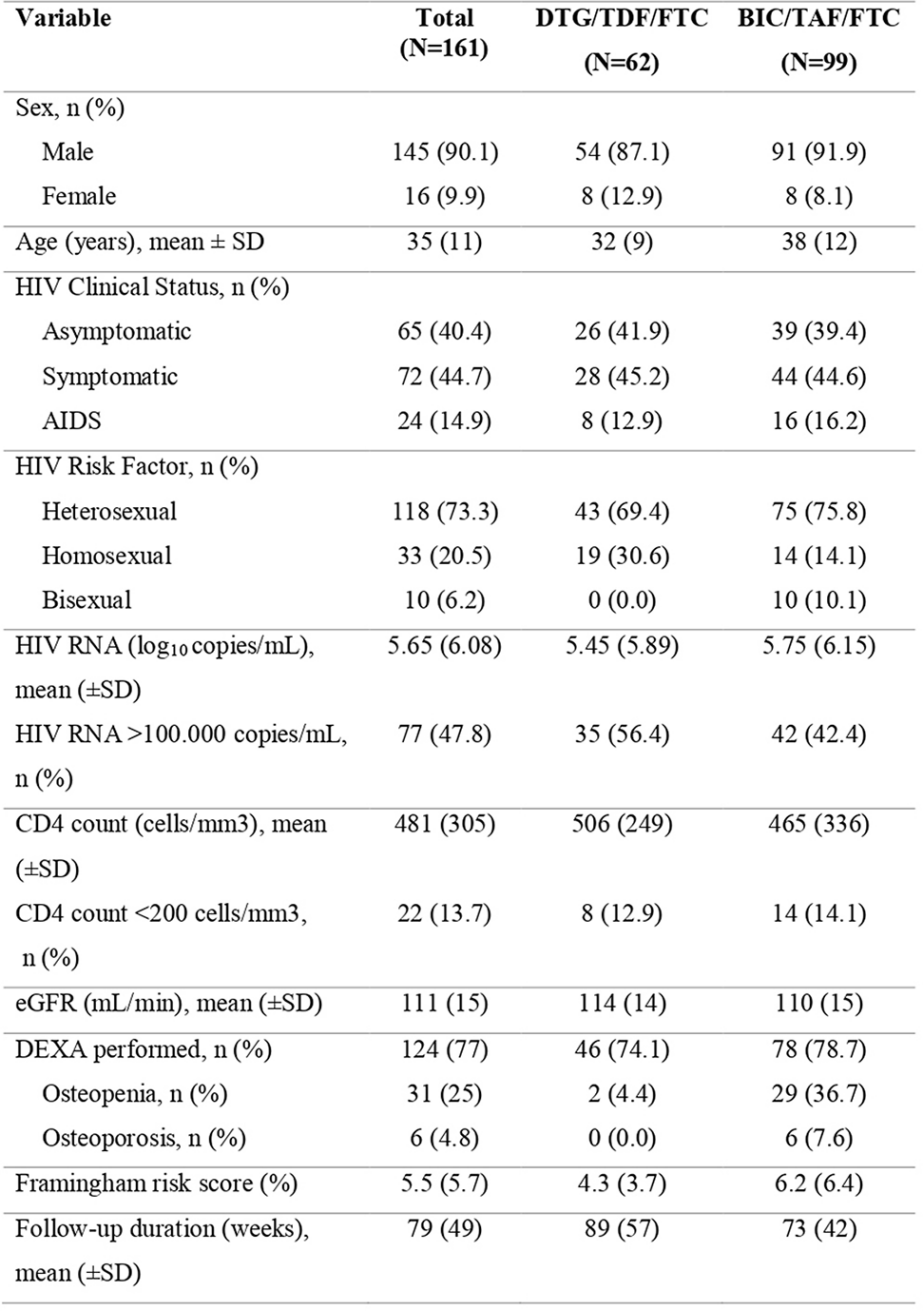
Baseline Demographic and Clinical Characteristics

**Figure:**
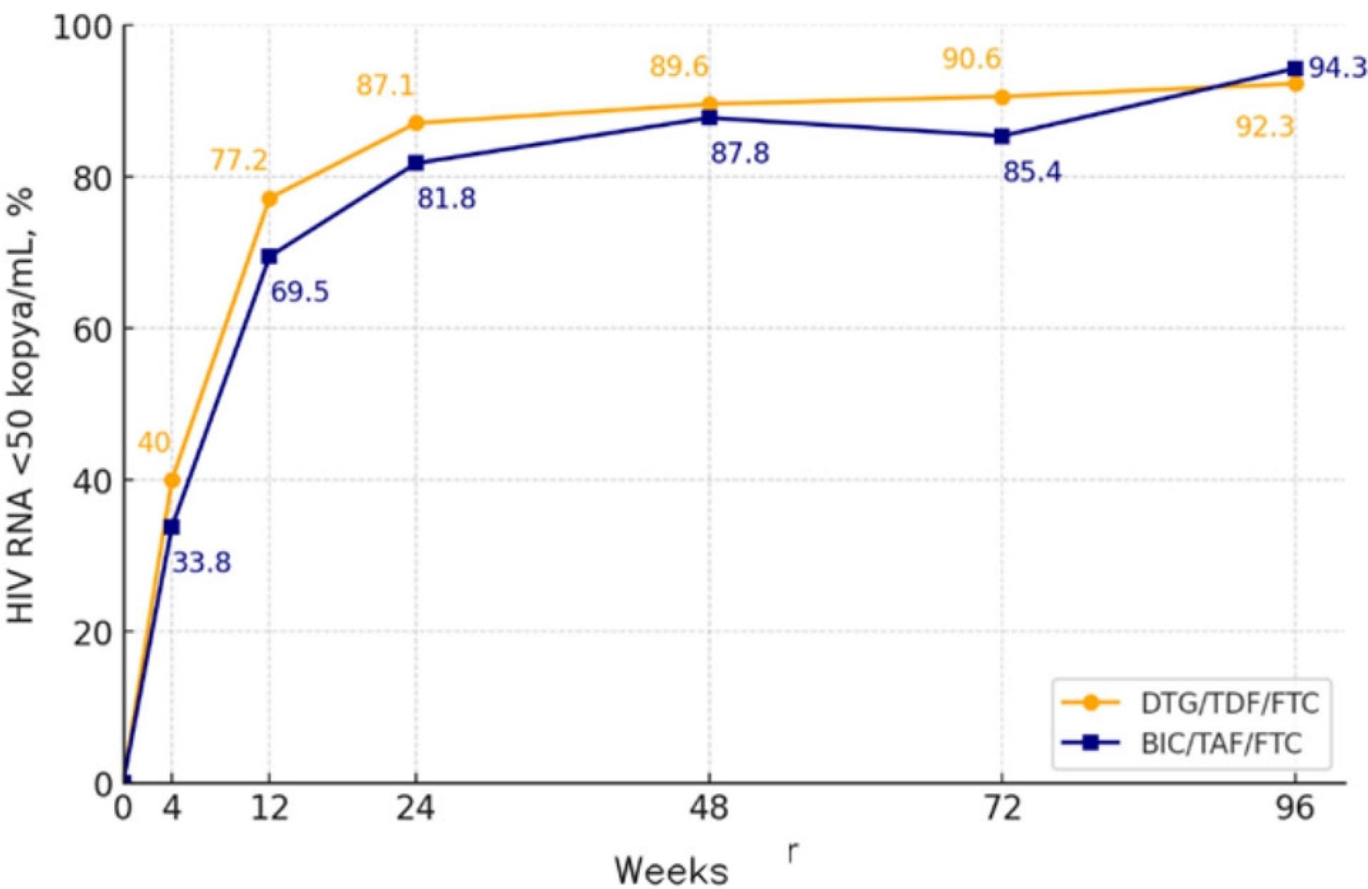
Changes in viral load following HIV treatment

**Methods:**

This retrospective observational study evaluated patients diagnosed with HIV between 2020 and 2024 who initiated either the BIC/TAF/FTC or DTG/TDF/FTC regimen and were followed for at least 24 weeks. Virological suppression, CD4 cell count increase, lipid profile, Framingham risk score (FRS) and adverse events were compared between the groups.

**Figure:**
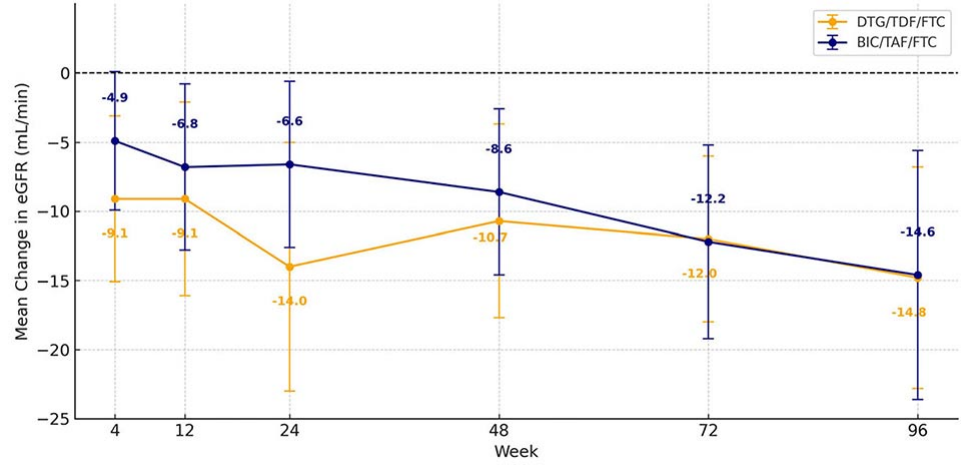
Changes in eGFR following treatment of BIC/TAF/FTC and DTG/TDF/FTC

**Results:**

A total of 161 patients were included (BIC/TAF/FTC: 99, DTG/TDF/FTC: 62). Virological suppression rates at weeks 24, 48, and 96 were high in both groups, with no significant difference (p=0.506, p=0.996, and p=1.000). At week 48, DTG/TDF/FTC showed 97.6% efficacy in patients with CD4 >200 cells/mm³ vs. 42.8% in those with CD4< 200 cells/mm³ (p< 0.001). In contrast, BIC/TAF/FTC showed 90% vs. 66.7%, respectively, but with no significant difference (p=0.073). BIC/TAF/FTC showed significantly better CD4 increases at weeks 48 and 96 compared to DTG/TDF/FTC, indicating better long-term immunological response (DTG/TDF/FTC: 161±157 and 182±149 cells/mm³; BIC/TAF/FTC: 289±234 and 395±256 cells/mm³; p=0.002 and p< 0.001). Although both groups showed decreases in eGFR levels, drug discontinuation due to nephrotoxicity was more frequent in the DTG/TDF/FTC group (12.9% vs. 0%, p< 0.001). In the FRS analysis for cardiovascular risk, a significant increase was observed in the BIC/TAF/FTC group over a mean of 1.6±0.6 years, while no significant change was detected in the DTG/TDF/FTC group over 1.9±0.8 years (p=0.019).

**Conclusion:**

Both regimens demonstrated high virological efficacy. BIC/TAF/FTC preserved efficacy in patients with low CD4 counts and showed a stronger long-term immunological response. However, the increase in lipid levels and FRS associated with this regimen should be monitored for long-term safety.

**Disclosures:**

All Authors: No reported disclosures

